# Relative letter-position coding revisited

**DOI:** 10.3758/s13423-021-02039-z

**Published:** 2022-01-19

**Authors:** Joshua Snell, Jonathan Grainger, Martijn Meeter

**Affiliations:** 1 Experimental and Applied Psychology, Vrije Universiteit Amsterdam, Amsterdam, The Netherlands; 2 CNRS et Laboratoire de Psychologie Cognitive, Aix-Marseille Université, Marseille, Provence France; 3 Vrije Universiteit Amsterdam, Amsterdam, The Netherlands

**Keywords:** Orthographic processing, Reading, Letter position coding

## Abstract

The notion that the brain achieves visual word recognition by encoding the relative positions of letters with open-bigram representations (e.g., ‘*h-e*’, ‘*h-r*’ and ‘*e-r*’ driving recognition of ‘*her*’) has been successful in accounting for many behaviors and phenomena. However, one characteristic of open-bigrams has remained unexplored: How is the activation of a bigram modulated by the distance between its constituents in the visual field? On the one hand, contiguous letters (e.g., ‘*at*’ in ‘*father*’) may allow for a clearer percept of the bigram. On the other hand, an increasing distance between letters (e.g., ‘*ae*’ in ‘*father*’) should create more certainty about their relative positions, which is precisely what the bigram is meant to convey. This matter was investigated with two experiments in which participants indicated whether target pairs of letters occurred in random letter strings. They were instructed that letter order mattered (i.e., ‘*a-b*’ does not occur in ‘*kbac*’), while letter contiguity did not (i.e., ‘*a-b*’ occurs in ‘*akcb*’). Controlling for crowding and eccentricity, bigrams were recognized faster upon decreasing the letter distance. However, when switching the target letter order (meaning the string should be met by a ‘*no*’ response), shorter letter distances yielded slower responses and more false positives. Neither relative position-coding models nor absolute position-coding models accommodate both these patterns at once. We discuss how a complete account of our effects may instead combine elements from both model types.

## Introduction

A longstanding debate in word recognition research concerns the question of whether the brain encodes the absolute or relative positions of letters (Davis, 2010a, 2010b; Gomez et al., 2008; Grainger & van Heuven, 2004; Kinoshita & Norris, 2013; McClelland & Rumelhart, 1981; Norris & Kinoshita, 2012; Snell, Bertrand, & Grainger, 2018a; Whitney, 2001, 2008). According to absolute position coding, individual letters would be associated with spatial locations (“*I see an ‘*h*’ at the first position and an ‘*e*’ at the second position; hence I see ‘*he”’). Relative position coding, on the other hand, states that the position of a letter is solely determined in relation to other letters (“*I see an* ‘h’ *on the left of an* ‘e’ *and an* ‘e’ *on the right of an* ‘h’; *hence I see ‘*he”’). Although these two classes of theory paint decidedly different pictures with respect to the cognitive architecture underlying reading, they have more or less provided equally good fits to the data. Consequently, neither hypothesis has been effectively falsified. The present study is to arbitrate between absolute and relative letter-position coding with a novel paradigm that sparks novel predictions.

### Bigrams and noise channels

Relative letter-position coding was conceived as a means to account for various behaviors that could not be explained if readers were to straightforwardly perceive letters in their canonical order. The lion’s share of these behaviors was observed in the masked priming lexical decision task (Forster et al., 1987; Forster & Davis, 1984). Broadly speaking, the extent to which a given string of letters primes a target word follows a graded function, based both on the number of shared letters between the prime and target, and on how many of those letters are correctly positioned in the prime. For instance, ‘*rcok*’ primes ‘*rock*’ to a greater extent than does ‘*radk*’, though not as much as the identical prime ‘*rock*’ (e.g., Andrews, 1996; Grainger, 2008; Perea & Lupker, 2004; Peressotti & Grainger, 1999). The facilitation by these so-called transposed-letter primes decreases as the distance between the transposed letters increases (e.g., ‘*kocr*’ primes ‘*rock*’ to a lesser extent than does ‘*rcok*’) (Perea et al., 2008; Perea & Lupker, 2004). The relative position coding account of these patterns involves bigram representations. The lexical representation ‘*rock*’, for instance, would be activated by neural clusters coding for bigrams ‘*ro*’, ‘*oc*’, ‘*ck*’, ‘*rc*’, ‘*ok*’ and ‘*rk*’ (e.g., Dehaene et al., 2005; Grainger et al., 2006; Grainger & van Heuven, 2004; Whitney, 2001, 2008). Most of these bigrams are also activated by the string ‘*rcok*’, while only one correct bigram is activated by the distant transposition string ‘*kocr*’ and the substitution letter string ‘*radk*’, hence explaining the weaker priming observed for the latter prime types.

An alternative account is provided by the notion of noisy slot-based position coding. This entails an extension of the classic Interactive Activation Model (McClelland & Rumelhart, 1981), according to which each constituent of a letter string is assigned a slot where it activates letter detectors dedicated to that slot. By these fundamentals alone, ‘*rcok*’ would provide no more evidence for ‘*rock*’ than would ‘*radk*’, a prediction robustly refuted by the data. The solution is to assume that a letter detector is sensitive not just to visual input aligning with the detector’s slot, but also to input aligning with surrounding slots, with activation diminishing as the number of intervening slots increases (Gomez et al., 2008; Norris et al., 2010). As such, a ‘*c*’ at position 2 would also provide some evidence for a ‘*c*’ at position 3, while an ‘*o*’ at position 3 would provide some evidence for an ‘*o*’ at position 2; hence causing stronger priming of ‘*rock*’ by ‘*rcok*’ than by ‘*radk*’.

A slightly different take on the notion of noise is provided by Norris and Kinoshita (2012). They propose that the brain does not have a coding scheme altogether. Instead, words would be perceived relatively directly from print, albeit only after passing through a ‘noise channel’. The noise channel would randomly insert or delete letters from the visual input, and would jumble the order of letters a bit. As such the noise channel accounts for the phenomena described above. However, the cognitive architecture of this noise channel is unspecified. It resembles a black box into which one may stow any desired cognitive operation (e.g., distorting input, adding random letters, removing random letters). As such the noise channel is unfalsifiable, and, one might contend, theoretically shallow.

That is not to say that the other frameworks (excepting the original IAM model) are easily falsified – especially given that they were all conceived a posteriori, guided by vast amounts of data. In terms of orthographic manipulations, virtually no stone has been left unturned in the masked priming paradigm, meaning that within the realm of this task, models do not produce novel predictions through which to arbitrate among theories.

It is therefore worth noting one alternative means to validate models, offered by the flanker paradigm. Studies have shown that the recognition of a word is impacted by the identities and relative positions of the constituents of flanking letter strings (e.g., Dare & Shillcock, 2013; Grainger et al., 2014; Snell, Bertrand, & Grainger, 2018a). Such influences between spatially distinct stimuli appeal to the idea of location-invariant orthographic representations integral to relative position-coding models (see also Marzouki et al., 2013, for priming effects with spatially distinct stimuli). In contrast, these patterns are more difficult to explain by means of a noisy slot-based model. As noted by Davis (2010b), allowing letter recognition to be influenced by stimuli that are so spatially distinct renders a slot-based model unable to distinguish even extreme anagrams such as ‘*bnoclay’* and *‘balcony’*.

### Probing bigram nodes directly

Having exposed a preference for relative position coding, we must here admit that several characteristics of the hypothesized bigrams have not been empirically verified. According to bigram models, the extent to which each individual bigram is activated by visual input depends on several things. Perhaps the most obvious factor is the visibility of each of the bigram’s constituent letters (Grainger & van Heuven, 2004; Snell, van Leipsig, et al., 2018b). Visibility is largely determined by acuity (i.e., each letter’s eccentricity from fixation) and crowding (affected by whether or not letters are on the word’s edge) (Tydgat & Grainger, 2009). Thus, the bigram ‘*te*’ would be activated to a larger extent by the stimulus ‘*tare*’ than by the stimulus ‘*tear*’, while the bigram ‘*ar*’ would be activated to a further extent by ‘*tear*’ than ‘*tare*’. Additionally, most bigram models assume that a bigram’s activation is weighted by the distance between the two letters in the visual field, with decreasing activation as the number of intervening letters increases (Grainger et al., 2006; Whitney, 2008), so that the bigram ‘*te*’ would be activated more by ‘*tear*’ than by ‘*toes’*.

Note that irrespective of which factors are assumed to modulate bigram activation, the summed activation of all bigrams never varies across strings of equal length.^1^ By mathematical consequence, any bigram model’s fit to the data in the masked priming task is realized largely by describing the number of shared bigrams between the prime and target (see, e.g., Schoonbaert & Grainger, 2004), not by specifying the contribution of each individual bigram per se. Precisely because the aforementioned paradigms probe word recognition rather than individual bigram recognition, it is the variations in individual bigram activation that have yet to be revealed empirically.

In the present study, we test the recognition of pairs of letters (i.e., bigrams) as a function of the distance between those letters. Importantly, although the default notion is that contiguous bigrams are more strongly activated than ‘open’ bigrams, this is not yet supported by neurophysiological or behavioral evidence (but see, e.g., Dehaene et al., 2005, for a discussion of constraint on bigram letter distance imposed by receptive field size). In fact, from a functional point of view, it would not seem odd to reason in the opposite direction, that is, stronger activation of the bigram as the number of intervening letters increases. The rationale here would be that there is more certainty about the relative positions of letters when they are further apart; and it is precisely the relative positions of letters that the bigram must convey. Clearly then, a test of the default assumption is due.

Crucially, with the inclusion of a condition with reversed target letters (e.g., “*does the target bigram ‘a-b’ occur in ‘cbad”* ?”), bigram models make different predictions about the outcomes of our experiments than do noisy slot-based coding models. Specifically, as the distance between two letters in the visual field decreases, a noisy slot-based model should increasingly see erroneous evidence for the letter pair in reversed order (so that the model wouldn’t spot the pair ‘*a-b*’ in ‘*bcra*’, but might erroneously spot ‘*a-b*’ in ‘*cbar*’). Thus, according to a noisy slot-based model, contiguous bigrams should be missed more often than open bigrams. When the target letters are reversed (so that the trial should be met by a ‘*no*’ response), a noisy slot-based model must have an easier time rejecting distant target letters than contiguous target letters.

Most bigram models, on the other hand, do not predict any effect of letter distance in the reversed letter condition, because the activation of the target bigram should be zero at all times (Grainger & van Heuven, 2004; Whitney, 2001, 2008). One exception is the Overlap OB-model of Grainger et al. (2006), which, akin to the Overlap model of Gomez et al. (2008), adopts positional uncertainty at the retinotopic level. In this model the target bigram would thus be activated to some extent by contiguous reversed letters.

In sum, here we report two experiments that, for the first time, directly probed the recognition of pairs of letters as modulated by the distance between those letters. Participants were instructed to indicate in each trial whether a target letter pair occurred in a subsequently presented string, whereby they had to pay attention to the order (e.g., ‘*a-b*’ does not occur in ‘*bcra*’). Using the above rationale, manipulating both the distance between target letters and the relative order of target letters allowed us to arbitrate between absolute and relative letter-position coding.

As the two experiments were identical in many respects, the *Methods* section covers both experiments.

## Methods

### Participants

For each experiment, 24 students (14 female; average age 20.5 years) from the Vrije Universiteit Amsterdam gave informed consent to participate for course credit. They all had normal or corrected-to-normal vision. None of Experiment 1’s participants participated in Experiment 2.

### Materials and design

As indicated before, the task was to indicate, on each trial, whether a target pair of letters occurred in a subsequently presented six-letter string. On each trial, six unique letters were randomly sampled from the alphabet (e.g., ‘*tsroma*’) in order to create the six-letter string. The target letter pair was determined by two experimental factors: the response condition (*target pair present, reversed,* or *absent*) and the distance between the two letters. In the *pair present* and *reversed* conditions, the target bigram could be made from any of the string’s 15 letter combinations (i.e., letters 1+2, 1+3, 1+4, …, up to letters 5+6), all with an equal number of occurrences. In the *pair absent* condition, the target letters were randomly picked from the alphabet’s remaining 20 letters. Per 15 trials of the *present* and *reversed* conditions (i.e., all possible letter combinations), the experiments comprised 15 trials in the *absent* condition. The *pair absent* trials were merely included to induce the task. All 45 conditions were tested ten times per participant. The 450 trials were run in random order.

Importantly, all six letter locations were equally likely to be occupied by one of the target pair’s constituents. This was done to ensure that participants would not develop an attentional bias to particular parts of the string. However, we planned to analyze only a subset of the position conditions, in order to avoid confounds of crowding and acuity. Consider, for instance, that in order to test the influence of bigram letter distance, one cannot simply compare the recognition of letters 3+4 (distance 1) to the recognition of letters 1+6 (distance 5), due to several imbalances: letters 1 and 6 suffer from less crowding, while letters 3 and 4 are viewed with better acuity.

Hence, although we had 15 position conditions, from these we analyzed a subset of eight experimental conditions, the remaining seven conditions being filler trials to prevent participants from focusing on specific letter positions. Four of the experimental conditions tested contiguous (short-distance) bigrams, while the other four tested non-contiguous (long-distance) bigrams. The crux was that overall crowding and acuity were perfectly equal between these two subsets of conditions (see Table 1).

**Table 1:** The eight experimental conditions retained for analysis, so that crowding and acuity were precisely equal for contiguous and non-contiguous bigram targets. Target letters are shown in bold. Total number of crowding letters refers to the summed numbers of letters adjacent to the target letters. For Total distance from central fixation we counted, from the center (i.e., in between the third and fourth letters), the summed numbers of letter spaces to the target letters

Contiguous bigram conditions	Non-contiguous bigram conditions	Total no. of crowding letters	Total distance from central fixation
**AB**cdef	**A**bcd**E**f	3	5
a**BC**def	a**B**c**D**ef	4	3
abc**DE**f	ab**C**d**E**f	4	3
abcd**EF**	a**B**cde**F**	3	5

### Procedure

**Fig. 1:**
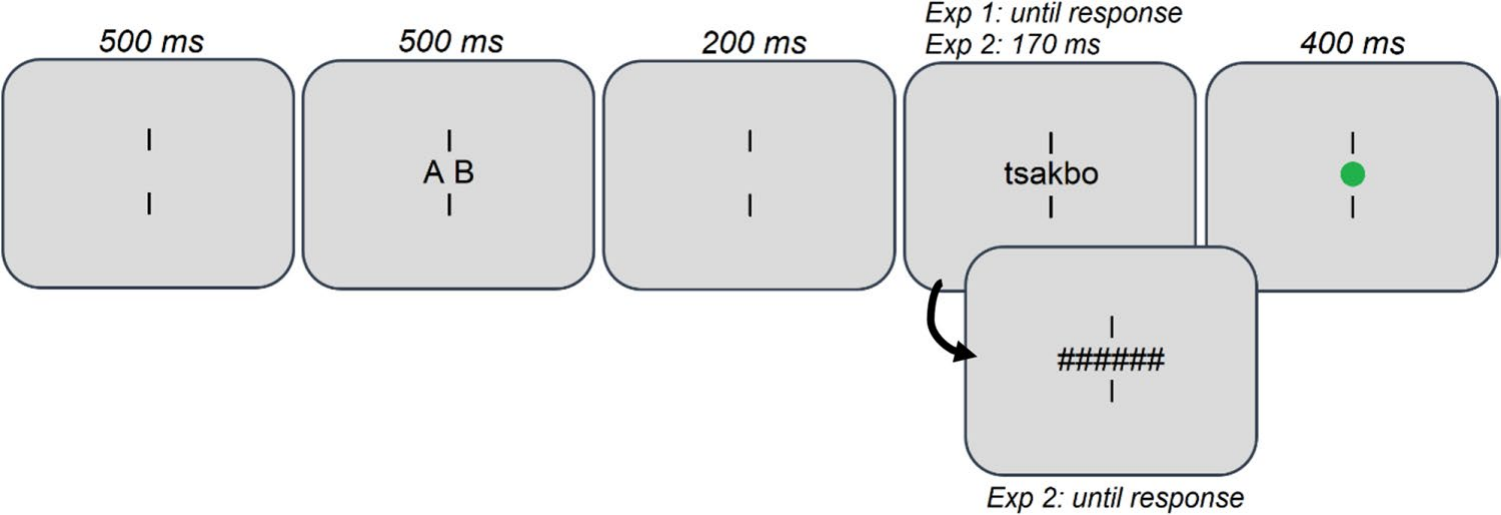
Example of the trial procedure. The size of stimuli relative to the screen is exaggerated in this example

A schematic representation of the trial procedure is provided in Fig. 1. Throughout the experiments, participants were instructed to maintain their focus on the center of the display, in between two vertical fixation bars. Each trial started with a fixation display, followed by a 500-ms presentation of a target letter pair at fixation. The target letters were presented in upper case and separated from one another by a single letter space to enhance visibility. The target letters were then replaced by a 200-ms fixation display, followed by a string of six letters, presented in lower case (hence minimizing the potential reliance on low-level visual cues). The sole difference between Experiments 1 and 2 was that the six-letter string remained visible until response in Experiment 1, whereas it was shown for only 170 ms and replaced by a mask (‘*######*’) in Experiment 2. As shall be reflected upon later, the shorter presentation duration in Experiment 2 was to discourage participants from scanning the letters serially. Participants had to indicate, by means of a left- or right-handed keyboard button press, whether the target pair was absent or present in the string, respectively. They were instructed that letter order mattered (e.g., ‘*a-b*’ does not occur in ‘*bcar*’), but that contiguity did not (e.g., ‘*a-b*’ occurs in ‘*carb*’). After the response, participants received feedback in the form of a green or red fixation dot for correct and incorrect answers, respectively. Prior to the 450 experimental trials, participants started with a 12-trial practice session. They were allowed a break halfway through the experiment. The experiments lasted 20–25 min.

## Results

In the analyses of response times (RTs), we only included correctly answered trials, belonging to either of the conditions listed in Table 1, with a reaction time (RT) within 2.5 SDs from the grand mean. Only the latter two criteria were applied for the analyses of errors.

RTs and errors were analyzed with linear mixed-effect models (LMMs) that included by-participant intercepts and slopes as random effects. Our key variable of interest was target letter contiguity (*contiguous* vs. *non-contiguous*), analyzed separately for *target present* and *target reversed* trials. We considered values of | *t* | (RTs) and | *z* | (errors) greater than 1.96 to be significant.

### Experiment 1

**Fig. 2:**
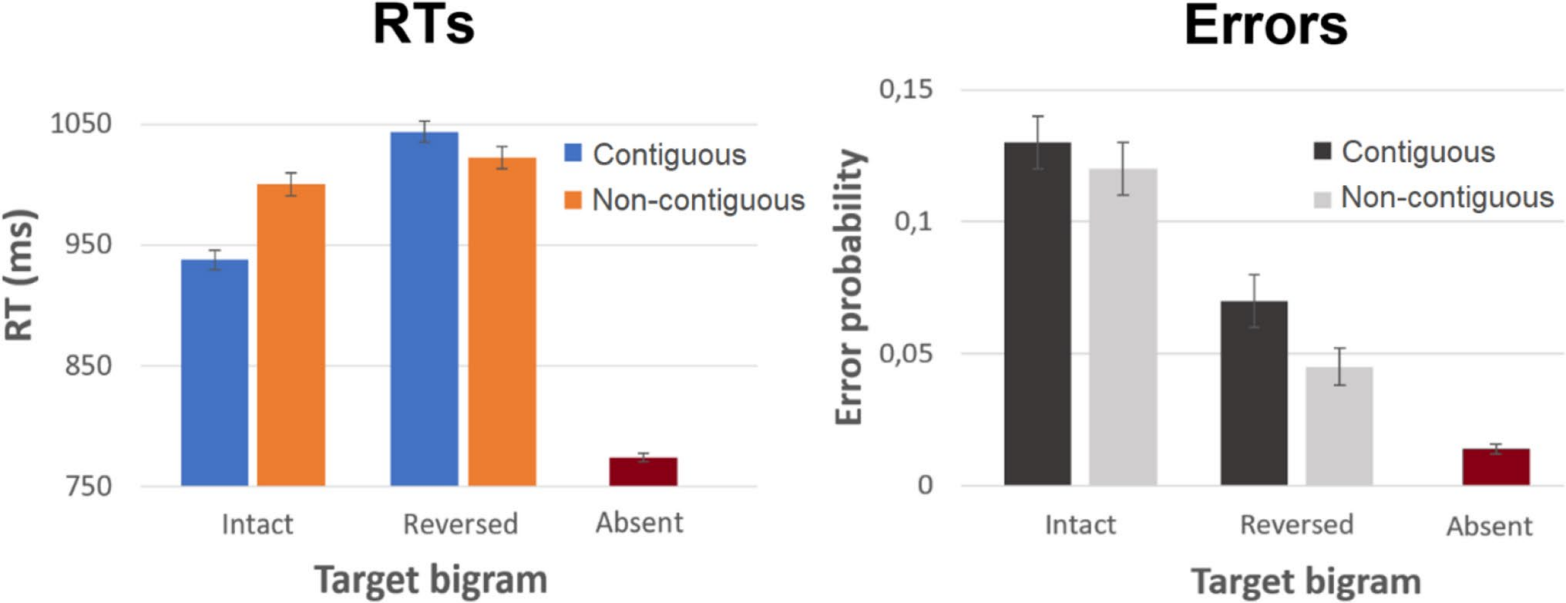
Experiment 1 results. Error bars represent standard errors. *RT* reaction time

In Experiment 1, where letters were visible until the participant’s response, contiguous target letters (presented in the correct order) were recognized significantly faster than non-contiguous target letters (*b* = -66.72, SE = 11.19, *t* = -5.96) (see Fig. 2). The error rate did not differ significantly between contiguous and non-contiguous target trials (*b* = 0.08, SE = 0.14, *z* = 0.58).

When presenting target letters in reversed order (so that the trial should be met by a ‘*no*’/ ‘*target absent*’ response), a reversed pattern of effects was observed. Contiguous reversed letters yielded a higher number of false positives (*b* = 0.44, SE = 0.22, *z* = 2.03), and this effect was accompanied by increased RTs for contiguous reversed target letters as compared to non-contiguous reversed letters. Note however, that the latter pattern was a mere trend (*b* = 20.67, SE = 11.76, *t* = -1.76).

Although direct comparisons between the intact and reversed bigram conditions are entirely tangential to the central investigation, it might be worth considering the apparent speed-accuracy trade-off whereby intact bigram trials were responded to faster, but with a higher error rate, than reversed bigram trials. This may have been caused by the fact that participants had to respond *‘no’* twice as often as ‘*yes*’, causing a response bias that negatively impacted the ‘*yes*’ trials. Again, note that our study does not warrant comparisons between intact and reversed bigrams in the first place.

According to the default open-bigram model, contiguous reversed letters should cause no more activation of the target bigram than should non-contiguous reversed letters (more precisely, both should provide zero evidence for the target bigram). Our results therefore led us to wonder whether the task, as implemented in Experiment 1, may have induced atypical letter-processing strategies. Specifically, participants may have performed a serial scan of the letter string (thereby possibly not relying on bigram representations), as opposed to the parallel processing of letters seen in normal word recognition (e.g., Adelman et al., 2010).

### Experiment 2

**Fig. 3:**
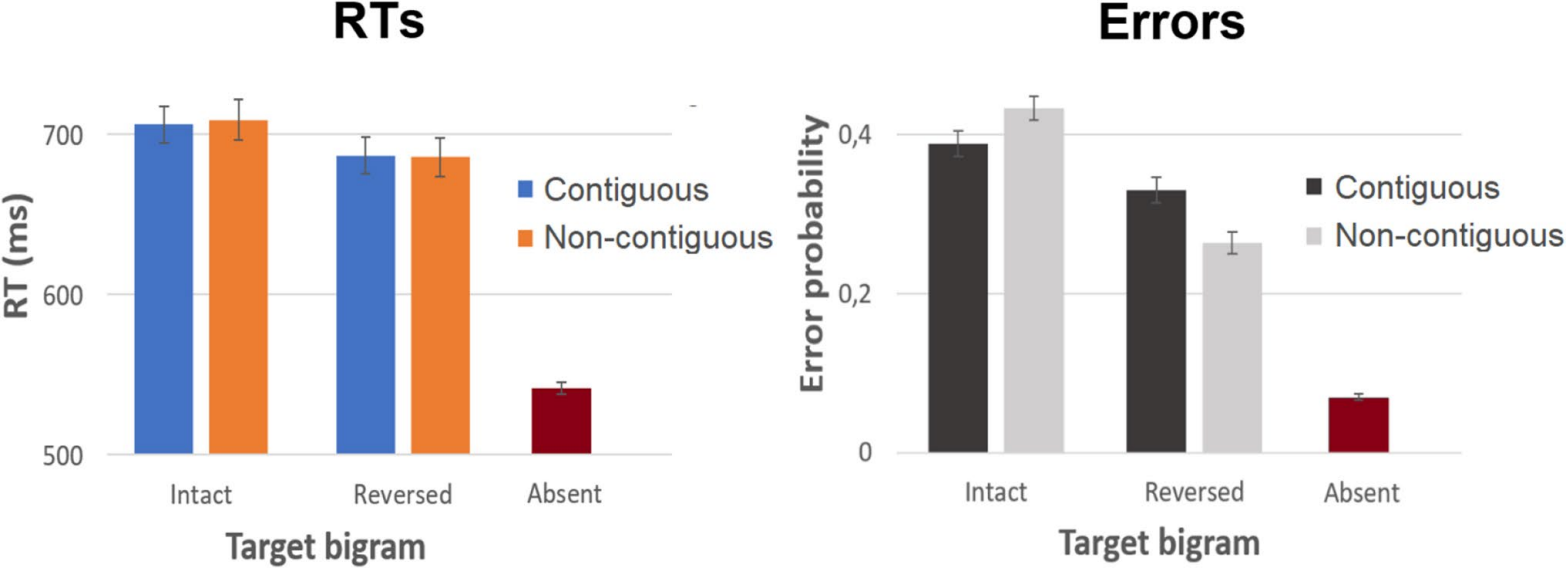
Experiment 2 results. Error bars represent standard errors. *RT* reaction time

To prevent participants from scanning the letters serially, in Experiment 2 strings were shown briefly (170 ms) and followed by a mask. This resulted in an absence of a difference in RTs between contiguous and non-contiguous target letters (*b* = -4.33, SE = 13.77, *t* = -0.32; see Fig. 3), but on the other hand a significantly higher number of misses in the case of non-contiguous target letters (*b* = 0.23, SE = 0.10, *z* = 2.25). Hence, in line with Experiment 1, we observed that an increasing distance between a target bigram’s constituent letters led to worse recognition. The absence of an effect in RTs (and concurrent higher error rate) in Experiment 2 compels us to believe that participants had to make partial guesses on the basis of limited visual input. In other words, whereas additional scanning time aided the recognition of non-contiguous bigrams in Experiment 1, there was no point in delaying the response in Experiment 2 given that the stimulus had already disappeared (hence the absence of an effect in RTs).

As in Experiment 1, patterns were mirrored for trials in which the target letters were reversed. Whereas again no effect was observed in RTs (*b* = -4.54, SE = 13.83, *t* = -0.33), contiguous reversed letters yielded significantly more false positives than did non-contiguous reversed letters (*b* = 0.36, SE = 0.11, *z* = 3.34).

## Discussion

In spite of decades of research, the question of whether the reading brain encodes the absolute or relative position of letters has not been definitively answered. Here we tested, for the first time, people’s ability to detect target pairs of letters embedded in letter strings, as a function of the distance between the target letters. In doing so, we made sure that crowding and acuity – known to be key factors in the ease of letter detection – were perfectly balanced across conditions. Minding letter order, absolute letter-position coding models (e.g., Davis, 2010a; Gomez et al., 2008; Norris et al., 2010) predict that performance should be best when target letters are further apart. After all, the closer together the target letters are, the more evidence these models should see for the target letters in reversed order (due to the positional noise assumed by all contemporary models), thus potentially yielding an incorrect response. Similarly, when target letters are presented in reversed order, (noisy) absolute position coding models should be more likely to see erroneous evidence for the target bigram upon decreasing the distance between target letters. The data from our two experiments are not entirely in line with these predictions. On the one hand, reversed target letters yielded more false positives when they were closer together; but then again, correctly ordered target letters did not yield more misses when being closer together.

Admittedly, the open-bigram model does not fare better in explaining our results. In line with patterns observed in the intact letter order conditions, the default take on open-bigrams is that contiguous letters must generate more activation of the bigram than non-contiguous letters (Grainger & van Heuven, 2004; Whitney, 2008) (note, however, that these models provide no mechanistic explanation for this). Yet, in these models reversed letters must provide zero evidence for the bigram, regardless of the distance between the letters: a prediction refuted by the present data. Later studies of relative position coding in fact alluded to the possibility that non-contiguous letters provide more activation, due to there being more certainty about the relative position of letters (Dandurand et al., 2011; Grainger et al., 2006; Snell, Bertrand, & Grainger, 2018a). But with such an approach (whereby one may account for the patterns observed with reversed letters), the model no longer accounts for the patterns observed with correctly ordered letters.

As we see it, our results invite an attempt to combine elements from both model classes. Specifically, a noisy absolute position-coding model that comprises, in addition to its single-letter detectors, bigram detectors, would effectively explain all our effects. Rather than comprising a discrete array of slots pertaining to single letters as the sole unit of representation, the brain may do with detectors of varying sizes and scopes. Very much appealing to statistical learning principles, the core idea is that the set of detectors, and the degree to which these detectors pertain to one or more symbols, is shaped by prior experience. Taking an extreme example, if a completely naïve reader were to encounter the ‘*h*’ solely in conjunction with the ‘*r*’, that completely naïve reader would have no means of knowing that ‘*hr’* in fact consists of two separate things, and thus it would appear unlikely that the reader would have discrete detectors for ‘*h*’ and ‘*r*’. Indeed, this reader is more likely to have a single detector sensitive to the bigram ‘*hr*’. If, on the other hand, the ‘*h*’ was encountered in conjunction with all letters except the ‘*r*’, the reader would be unlikely to have developed a detector for ‘*hr*’, while the development of a detector for the ‘*h*’ as a discrete unit appears sensible. Naturally, the sum of our prior experiences aligns with neither of these extremes, but instead constitutes a vast continuum in between the two. From here it is not difficult to conceive that readers may have both single- and multi-letter detectors.

We recognize that such theorizing complicates the slot-based nature of most absolute position-coding schemes (excepting the spatial coding approach of Davis, 2010a, 2010b). For instance, it is unclear whether a slot would accommodate both single letters and bigrams, or whether bigram detectors would occupy two slots at once, etc. In this regard, it is worth noting a recent proposal by Snell, Bertrand, and Grainger (2018a), which would render the entire concept of a slot obsolete. Snell, Bertrand, and Grainger (2018a) reasoned that the brain could deduce the ‘leftness’ and ‘rightness’ of letters by the activation that they generate in each of the brain’s respective hemispheres. For instance, a letter situated far away in the right hemifield would generate weak bottom-up activation (due to low visual acuity) in the left hemisphere and no bottom-up activation in the right hemisphere, and may thus be ‘interpreted’ by the brain as being in the right peripheral hemifield. A letter in the left visual hemifield would similarly generate activation in the right hemisphere as a function of acuity (and thus distance from fixation). This mechanism for estimating object locations applies as easily to bigrams as to single letters.^2^

A final remark must be made about the nature of this novel task, and the extent to which it may or may not appeal to the cognitive mechanisms underlying reading. One might contend that the task is so artificial in nature that the brain is not compelled to engage in orthographic processing (rather, the brain might be engaged in, say, object scanning in sensory or working memory). In this regard it is worth noting the field’s rich history in the employment of single- and multi-letter detection tasks, and the established relationships between such tasks and natural reading proficiency (e.g., Chanceaux & Grainger, 2012; Geiger et al., 2008; Legge et al., 2001). It is our view that if the brain does indeed encode bigrams, these must be the brain’s obvious representation of choice in the execution of this task, irrespective of whether the task itself is a reading task per se. We may nonetheless conceive of a future line of study in this regard. Experimental designs akin to the one employed here may make use of words instead of random letters. In addition to recognizing target bigrams, participants might, in a dual-task setup, be instructed to make semantic decisions about words. As such one would ascertain that participants are engaged in word recognition, and thus that the mechanisms hypothesized here, if they indeed exist, are necessarily in play.

In conclusion, having put established models of letter position-coding to the test with a new paradigm that sparked new predictions, we were compelled to advocate against all individual frameworks as they currently stand, and instead to propose a hybrid model that encodes – through inferring the ‘left-’ and ‘rightness’ of stimuli (e.g., Snell, Bertrand, & Grainger, 2018a) – the absolute positions of both mono- and multi-gram representations.

## Footnotes

1 Top-down (e.g., word-to-letter) feedback connections not taken into account.

2 It may be noted that several phenomena in letter-position coding occur in marquee format (with vertically aligned letters) too. Yet, the present account relies heavily on the brain’s hemispheric organization. We therefore predict that the presently reported phenomena will not occur in marquee format.
